# Microbiome distribution modeling using gradient descent strategies for mock, *in vitro* and clinical community distributions

**DOI:** 10.1371/journal.pone.0290082

**Published:** 2023-08-21

**Authors:** Juan Ricardo Velasco-Álvarez, Nimbe Torres y Torres, Isaac Chairez, José Luis Castrejón-Flores

**Affiliations:** 1 Instituto Politécnico Nacional, Unidad Profesional Interdisciplinaria de Biotecnología, Gustavo A. Madero, Mexico City, Mexico; 2 Departamento de Fisiólogía de la Nutrición, Instituto Nacional Ciencias Médicas y Nutrición(“Salvador Zubirán”, Tlalpan, Mexico City, Mexico; 3 School of Engineering and Sciences, Técnologico de Monterrey-Campus Guadalajara, Zapopan, Jalisco, Mexico; University of Idaho, UNITED STATES

## Abstract

The human gut is home to a complex array of microorganisms interacting with the host and each other, forming a community known as the microbiome. This community has been linked to human health and disease, but understanding the underlying interactions is still challenging for researchers. Standard studies typically use high-throughput sequencing to analyze microbiome distribution in patient samples. Recent advancements in meta-omic data analysis have enabled computational modeling strategies to integrate this information into an *in silico* model. However, there is a need for improved parameter fitting and data integration features in microbial community modeling. This study proposes a novel alternative strategy utilizing state-of-the-art dynamic flux balance analysis (dFBA) to provide a simple protocol enabling accurate replication of abundance data composition through dynamic parameter estimation and integration of metagenomic data. We used a recurrent optimization algorithm to replicate community distributions from three different sources: mock, *in vitro*, and clinical microbiome. Our results show an accuracy of 98% and 96% when using *in vitro* and clinical bacterial abundance distributions, respectively. The proposed modeling scheme allowed us to observe the evolution of metabolites. It could provide a deeper understanding of metabolic interactions while taking advantage of the high contextualization features of GEM schemes to fit the study case. The proposed modeling scheme could improve the approach in cases where external factors determine specific bacterial distributions, such as drug intake.

## 1 Introduction

The human gut microbiome is a complex and dynamic microbial community found in the gastrointestinal tract, generally composed of bacterial members from the Firmicutes, Bacteroidetes, Actinobacteria, Proteobacteria, Fusobacteria, and Verrucomicrobia phyla [1], which is primarily affected by internal and external factors including age and gender or diet and medication [2, 3]. New generation sequencing and meta-omic (metagenomic, metatranscriptomics, metaproteomics, and metabolomics) techniques have allowed us to better understand the interaction and cross-talk among community members in this complex consortium, at the genetic and molecular level, including their change during health and disease. For instance, it has been suggested that the interaction between certain microbiome strains with the host can influence metastatic processes in colon-rectal cancer patients [4]. Even though there is an important association in the pathogenesis of several diseases, the underlying comprehension of bacterial ecology and interplay within the host is very complex. Therefore, it is not clearly understood [5, 6]. Recently, computational models often paired with statistical tools have been used to analyze and predict microbial community behavior from high throughput sequencing and omics of *in vitro* or *in vivo* analysis [7–9].

The most common computational models are ecological models based on the generalized Lotka-Volterra (gLV), particularly useful when abundant data are available. This approach utilizes ordinary differential equations in an unstructured strategy assuming that community stability depends on intrinsic species growth dynamics and classical ecological interactions (i.e, mutualism (++), commensalism (+0), amensalism (-0), parasitism (+-) and competition (–) [10]) with other microbial species. This reductionist approach elucidates some community behavior at a macro scale while most underlying metabolic interactions remain uncertain. Another modeling strategy, known as genome-scale metabolic modeling, utilizes metabolic network reconstructions of microbial species and integrates microbial interactions allowing cross-feeding between species inside the nutritional space for the simulation. Metabolic networks are created from the annotated genome sequences of a single species and converted into a mathematical stoichiometric matrix, where rows describe individual metabolites and columns describe metabolic reactions. These are analyzed using flux balance analysis (FBA), where a linear programming optimization problem is solved by maximizing an objective function subject to a set of constraints [11]. Typically, a steady state assumption is made where for each intracellular metabolite, the sum of all fluxes producing the metabolite is equal to the sum of all fluxes consuming the metabolite [12]. However, several dynamic FBA tools have been developed to utilize a quasi-steady state assumption to calculate metabolic activity at each time-step of a dynamic simulation [13–15]. Other existing frameworks, such as MICOM [16] and d-OptCom [17], facilitate the integration of metagenomic sequencing data with Genome-scale Metabolic Modeling (GEM).

In this study, we introduce a novel computational framework that deviates from existing approaches to implement a hierarchical gradient descent optimization algorithm, enabling accurate replication of abundance data from three distinct sources: mock, *in vitro*, and clinical abundances. Our framework leverages state-of-the-art metabolic modeling tools, such as the numerical simulator *μbialSim* [14]. It employs the optimization algorithm to modify dynamic parameters governing the community distribution. The objective is to attain the desired microbial composition in genome-scale metabolic model(GEM) simulations. The method we explored in this study provides an alternative approach that considers the biological interactions between microbial strains while promoting a more equitable representation of their relative abundance.

## 2 Materials and methods

### 2.1 Modeling strategy and hierarchical distribution of data

The first stage of the present work comprises the construction of a data-driven modeling technique based on applying two recurrent nested algorithms. The algorithms were implemented and executed in Matlab R2020b. The first algorithm is based on the dFBA modeling method, implemented using the COBRA toolbox [13] and μbialSim [14], which provides access for all simulated species to the pool of compounds, leading to the predominant interaction between microorganisms to be competition for these compounds and the production of secondary metabolites that may impact their behavior. While interactions such as quorum sensing or space competition may affect microbiome dynamics, they are not accounted for in the simulations.

The selected numerical simulator also permits the input of kinetic parameters to contextualize the microbial community simulation further. We took advantage of this feature to add an optimization algorithm to modulate kinetic constants for the simulation to obtain the desired microbial distribution at the end of the simulation. This is proposed as an alternative method to address complex disease-related contexts where kinetic data is scarce. The optimization algorithm utilizes iterative gradient descent.

**Fig 1:**
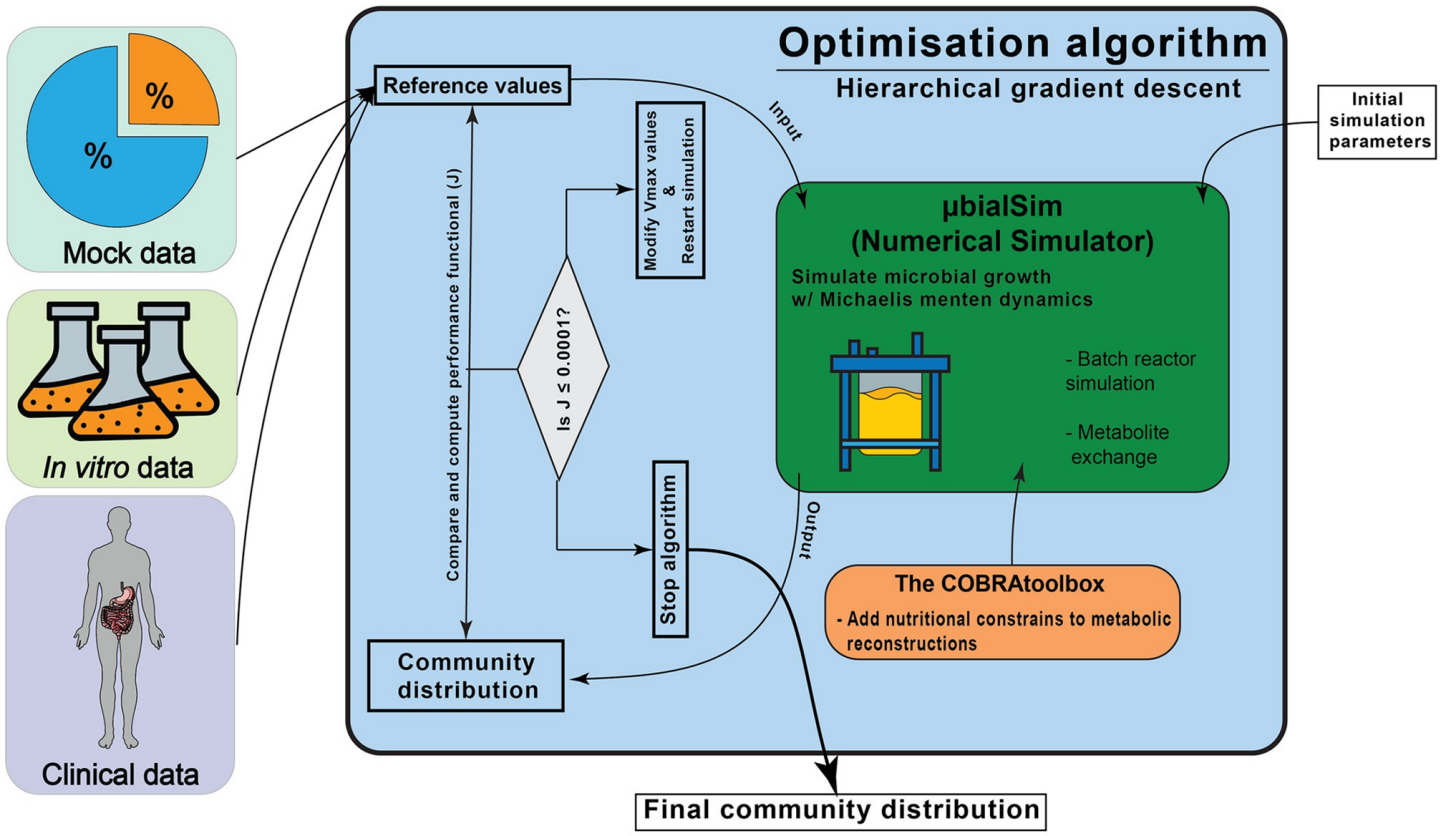
Proposed modeling strategy. General operation diagram of the modeling strategy considered in this study.

The suggested adaptive modeling implementation allowed us to use relative abundance data of microbiome communities as a reference point for the output of the dFBA simulation. This was achieved by modifying the *V*_*max*_ value for the dFBA in each iteration cycle until the reference composition is reached at the end of the simulation. A hierarchical modeling strategy was applied to avoid the loss of activity of the species with less proportion, in contrast to more represented strains, in complex community distributions. Each relative abundance profile was split into groups of representative strains (shown as bullet groups in Table 1). The reference values were then adjusted to normalize the proportional composition, and numerical simulations were performed for each group independently. This regrouping stage of the results provides a close understanding of the metabolic profile for the community. However, this may exclude interactions between under and over represented strain groups, which may be necessary. This could be solved if such interplay is previously known and considered in the group-splitting process. Simulation data of each group were again normalized and merged with the rest of the data to create a simulated community profile for each case (Fig 1), considering the proportional fraction for each species within each group in the microbial relative abundance profile. The proposed approach enhances the optimization results without altering crucial interactions as the biomass evolution remains unrestricted by the splitting step.

**Table 1:** Microbial strains for each of the cases used in testing the proposed modeling strategy.

Mock data	*in vitro data*	Clinical data
● *Akkermansia muciniphila*	● *Bacteroides fragilis*	● *Faecalibacterium prausnitzii*
● *Bacteroides fragilis*	● *Anaerostipes caccae*	● *Prevotella copri*
● *Bacteroides thetaiotaomicron*	● *Bacteroides thetaiotaomicron*	● *Bacteroides uniformis*
● *Coprococcus eutactu*s	● *Bacteroides caccae*	● *Akkermansia muciniphila*
● *Faecalibacterium prausnitzii*	? *Dorea longicatena*	● *Bacteroides caccae*
● *Prevotella copri*	➤ *Coprococcus comes*	● *Bacteroides fragilis*
● *Prevotella stercorea*	➤ *Bacteroides ovatus*	➤ *Ruminococcus bromii*
● *Ruminococcus bromii*	➤ *Bacteroides cellulosilyticus*	➤ *Bacteroides ovatus*
	➤ *Bifidobacterium pseudocatenulatum*	
	➤ *Eubacterium rectale*	
	❙ *Collinsella aerofaciens*	
	❙ *Dorea formicigenerans*	
	❙ *Prevotella copri*	
	❙ *Clostridium asparagiforme*	
	❙ *Bacteroides vulgatus*	
	✓ *Bacteroides uniformis*	
	✓ *Bifidobacterium adolescentis*	
	✓ *Clostridium hiranoni*s	
	✓ *Eggerthella lenta*	
	✓ *Desulfovibrio piger*	
	✓ *Parabacteroides johnsonii*	
	✽ *Faecalibacterium prausnitzii*	
	✽ *Bacillus cereus*	
	✽ *Blautia hydrogenotrophica*	
	✽ *Roseburia intestinalis*	

*Bullet points in the table represent the groups in which the community simulations were split to implement the hierarchical strategy for each dataset.

### 2.2 Testing the modeling scheme with data from three different sources

As shown in Fig 1, we evaluated the constructed computational modeling framework with three sets of reference values from previous studies. For each case, each species’ metabolic reconstructions for the different abundance profiles were obtained from the *AGORA* library of reconstructions [18] and retrieved from the Virtual Metabolic Human Database [19]. The flux boundaries of these reconstructions were modified using the tools provided by the COBRA toolbox to include the range of available nutrients within the simulations. Further details on the nutritional input for each simulation are mentioned in the following sections.

### 2.3 Modeling scheme capabilities to replicate a mock microbiome distribution

A mock community was created to establish baseline parameters for the presented modeling strategy. Eight representative gut microbiome species were selected. Initially, we selected an arbitrary reference abundance where each strain was equally represented in fractional abundance. However, the simulations demonstrated that this composition was not feasible due to biological constraints in the microbial consortia numerical simulation. To address this issue, we chose the resultant community abundance composition with the lowest J value as the simulation reference (Section 2.6.2). These reference values were previously observed to be possible in the simulations, allowing us to assess the replicability and validity of the modeling strategy. The optimization algorithm was then run again with this new set of simulations until the stopping criterion (Section 2.6.2) was satisfied. The nutritional space conditions used in the simulations were chosen to reflect an unhealthy diet, characterized by high lipid (49%), carbohydrate (30%), protein (16%), and alcohol (5%) content, as described in the Virtual Metabolic Human Database [20].

### 2.4 *In vitro* and clinical microbiome community distribution datasets and modeling scheme

For the *in vitro* and clinical instances, previously published bacterial distributions were selected as reference values for the optimization algorithm. For the *in vitro* case [21], the authors created a model to infer the effect of microbial interactions in butyrate production. The model utilized a consortium of 25 microbial species specifically selected for our study, in an attempt to replicate a microbial composition based on experimental data and model predictions (Fig 1). For this simulation, we used the chemically defined medium “DMR38” as reported in previous studies [21]. The concentrations of the chemical components were initially provided in mg/mol and were subsequently converted to mmol concentration to be used as nutritional input in the simulations (S1 Table). In the case of clinical data, the selected abundance reference values were reported previously [20]. The relative abundance representing a healthy Mexican patient’s gut microbiome was reported in that work. The authors obtained the profile using *Ilumina* sequencing technology. We selected eight bacterial strains that constitute the top 90% bacterial distribution representatives (Fig 1), to be used in the simulation as the reference distribution. This choice may result in omitting crucial metabolic inputs from low-abundance microorganisms. However, it should be noted that metabolic reconstructions for certain strains found in the clinical or experimental samples were unavailable in the *AGORA* database. The applied nutritional restriction was the same diet as the one used with the mock distribution test. The diet used in our study was selected to replicate the macro molecule concentration profile observed by [20]. The diet composition is the same as in Section 2.3.

### 2.5 Metabolite profile evolution for each of the study cases

Metabolite compounds produced and consumed by the simulated community in a specified nutritional state can be observed using the *μbialSim* simulator tool [14]. The reported metabolic profiles were generated using the trajectory obtained from the simulation at the end of the run. A set of eight metabolites were selected for analysis in this study. The metabolites were all chosen for their importance as molecules affecting the immune system [22, 23]. The complete breakdown of metabolite flux for each test can be found in the trajectory file generated during the simulation using μbialSim for microbiome community simulation. The trajectory files for each test in this paper are present in the S1 Dataset. The metabolites analyzed in this study included Acetate, Butyrate, Propionate, Formate, Indole, and Indole-3-propionate.

### 2.6 Modeling strategy

Inspired by the model-free optimization tools and reinforcement learning techniques, this study takes advantage of the biomass distribution information (obtained by genetic sequencing) and its relationship to the estimated states from the *μBialSim*/COBRA numerical evaluation to derive a data-driven model of microbiome distributions with a different number of microbes components. The state of the system representing the microbiome distribution is given by (*X*, *C*), where *X* = (*x*_1_, …, *x*_*n*_) defines the biomass concentration (in *gDW*/*L*) of the *n* microbial strains and $C_{j}=\left(c_{j,1},\ldots ,c_{j,m_{j}}\right)$ refers to the concentrations (in mM) of *m*_*j*_ pool compounds produced by the corresponding microorganism *x*_*j*_.

Selecting the relevant metabolites for the specific application depends on the metabolic network modeling goals. For example, metabolites assumed never to be growth-limiting can be ignored. Also, the modeling strategy should consider compounds for which experimental data are available.

The system dynamics are modeled using two ordinary differential equations. The specific microbial dynamics for the particular species *j* are provided by

$$\begin{array}{c} \frac{d}{dt}x_{j}=(x_{j}^{in}-x_{j})\frac{q}{V}+\mu_{j}x_{j} \end{array}$$ (1)

Where the microbial concentration in the inflow $x_{j}^{in}$ (*gDW*/*L*), flow rate *q* (*L*/*h*), culture volume in the reactor *V* (*L*), and specific growth rate *μ*_*j*_ (1/*h*). The dynamics of pool compound *i* in the bioreactor are given by

$$\begin{array}{c} \frac{d}{dt}c_{j,i}=(c_{j,i}^{in}-c_{j,i})\frac{q}{V}+\sum_{k=1}^{K_{j}}\sum_{l=1}^{L_{i}}S_{i,k,l}\times v_{l,R_{j}}\times x_{j} \end{array}$$ (2)

Here the inflow concentration is defined by $c_{j,i}^{in}$ (*mM*) that connects the *j* − *th* strain with the corresponding compound *c*_*j*,*i*_. Notice that the total concentration of this component is $c_{j}=\sum_{i=1}^{I_{j}}c_{j,i}$ where *I*_*j*_ is the number of strains that produce such a compound. The components *S*^*i*, *k*, *l*^ define the stoichiometry relations between the selected biomass *x*_*j*_ that produces the component *c*_*j*,*i*_ through the reaction rate constant $v_{l,R_{j}}$. This value also defines the direction of the exchange reaction, with the reaction proceeding in the forward direction, indicating metabolite excretion with a positive value at the corresponding value and metabolite uptake with a negative value. The flux of the exchange reaction corresponds to $v_{l,R_{j}}$ (*mmol*/*gDW*/*h*), which is the *i* − *th* reaction of the *j* − *th* species. The variable *K*_*l*_ represents the number of coupled exchange reactions for specie numbered with index *j* corresponding to the stoichiometry related to the reaction rate $v_{l,R_{j}}$. The value of these rates relates the specific stoichiometry with index *l* and the microorganism labeled with *j*. The variable *R*_*j*_ records the reaction IDs of the respective exchange reactions.

The values of the specific growth rates *μ*_*j*_ and exchange fluxes $v_{l,R_{j}}$ are derived with the solution of the FBA problem for each species individually. To solve this problem, the detectable compound concentrations in the bioreaction systems must be interpreted regarding their maximal allowable uptake rates. This is commonly obtained considering Monod-type kinetics. For the *i* − *th* exchange reaction of species *j* which is coupled to pool compound *C*_*j*,*i*_, the current reaction rate is given by:

$$\begin{array}{c} v_{l,R_{j}}=V_{max_{L,R_{j}}}\frac{S_{l,R_{j}}}{Ks_{l,R_{j}}+S_{l,R_{j}}} \end{array}$$ (3)

Here $V_{max_{L,R_{j}}}$ (*mmol*/*gDW*/*h*) is the maximum reaction rate constant which is dependent on a preferable substrate compound $S_{l,R_{j}}$. The constant value defined by $Ks_{l,R_{j}}$ characterizes the preference of each microorganism strain for the selected substrate $S_{l,R_{j}}$. For this study, and considering the parametric sensitiveness, the values of $Ks_{l,R_{j}}$ are fixed to constant scalars. Notice that here we have not considered complex forms of the reaction rate constant, which have been gathered in the value of $V_{max_{L,R_{j}}}$. Within each process of flux analysis in the traditional metabolic fluxes, the problem to be solved corresponds to the solution to the following optimization problem.

$$\begin{array}{c} \begin{array}{cccc} \text{minimize} & & C^{⊤}v\\ \text{subject}\;\text{to} & & Sv=b\\ & & & v_{lb}\leq v\leq v_{ub} \end{array} \end{array}$$ (4)

where *C* is a vector of linear objective coefficients, *S* is the matrix of stoichiometric coefficients for the molecular species involved in the considered reactions. The vector *v* is formed with all the values of $V_{max_{L,R_{j}}}$. To determine the general vector with bounded lower and upper bounds, a parametric identification algorithm is employed to estimate the appropriate values. It should be noted that while the optimal objective value is always unique, the optimal vector solution is often non-unique, meaning there can be multiple optimal solutions.

#### 2.6.1 Dynamic numerical simulation

The evaluation of microbial growth and compound production was evaluated using the COBRA Toolbox (V. 1.0) in the MATLAB software (Release 2020b) [13, 24]. The integration algorithm for the μBialSim is the *augmented forward Euler method* [14]. The time step size was fixed to Δ*t* = 0.1 h. This choice results from the numerical sensitivity to the adjustment method of parameters related to the maximum reaction rate. Using five parallel processors to improve the simulation performance, a parallel computing strategy was considered. The parameters of the integration method were: Accuracy = 1*10^−9^, Bio-Accuracy 1*10^−9^, Steady State Accuracy = 1*10^−2^, maximum Deviation = 25.0, biomass Reduction Factor = 8.0 and record Limiting Fluxes = 1.0 [14]. The rest of the *μBialSim* parameters were used as the toolbox suggests. This numerical strategy was developed in a laptop with Intel Core i7 2.2 GHz, 16 GB DDR4 RAM.

The initial concentrations of microorganisms biomass (forming the inoculum) were all fixed to 0.1 g/L to remove any bias between the different strains in the evaluated microbiome. All the simulations were performed using these values, except when some particular microorganism in the microbiome did not exhibit any growth. For such cases, the initial concentration was fixed to the biomass concentration obtained in the final step of the exploratory simulations, according to the particular instance. The nutrient concentration of the culture broth was defined according to each numerically evaluated case considering the artificial or reported culture broth (see Material and methods).

To keep the biological sense of the simulation, all the biomass concentrations were limited to the range [0, *X*^*max*,*i*^] where *X*^*max*,*i*^ is the maximum theoretical value of each strain in the studied microbiome. For this study, all these values were fixed to *X*^*max*,*i*^ = 50 g/L, thus limiting the growth of faster species and reaching a persistent excitation condition for the system. The value of *K*_*s*_ was fixed to a given value of 0.10, as indicated by [14]. Nonetheless, both parameter values can be obtained from literature and used as needed on the model.

#### 2.6.2 Gradient descent optimization algorithm

Considering the equation:

$$\begin{array}{c} J_{k}=\sum_{i=1}^{N}{\left(\frac{1}{X_{k}}x_{i,k}-\frac{1}{{\hat{X}}_{k}}{\hat{x}}_{i,k}\right)}^{2} \end{array}$$ (5)

where $X=\sum_{i=1}^{N}x_{i}$ is the total biomass concentration determined for each experimental scenario, and *x*_*i*_ is the biomass concentration for each strain in the microbiome. In addition, ${\hat{X}}_{k}=\sum_{i=1}^{N}{\hat{x}}_{i,k}$ is the total estimated biomass after the *k* − *th* cycle in the reinforcement learning, and ${\hat{x}}_{i,k}$ is the corresponding estimated biomass for each strain in the simulated scheme. The value *J*_*k*_ qualifies the modeling strategy of the estimated aggregated biomass fraction. An early stop criterion for the algorithm was implemented where the value of *J*_*k*_ had to reach 0.001. Otherwise, the code was manually stopped if the function reached a local minimum.

## 3 Results

The simulation workflow is constructed based on the μBialSim toolbox with the addition of the recurrent nested gradient descent algorithm. The steps involve (1) selecting the microbiome species, (2) retrieval of metabolic reconstructions for such species from the *AGORA* database [18], (3) selecting and implementing nutritional restrictions in metabolic reconstructions, (4) initially setting simulation parameters for the gradient descent algorithm (i.e., initial biomass concentration per species, initial *Vmax*, *ks*, and reference composition values) and (5) executing the code. This section details the outcome of the three examples we tested for the proposed modeling strategy using the recurrent gradient descent methodology. The data in the following sections present the results proportionally merged as a single system in the cases where the hierarchical strategy is applied (Fig 1, Section 2.1).

### 3.1 Evaluating the optimization algorithm with data from a mock microbiome

**Fig 2:**
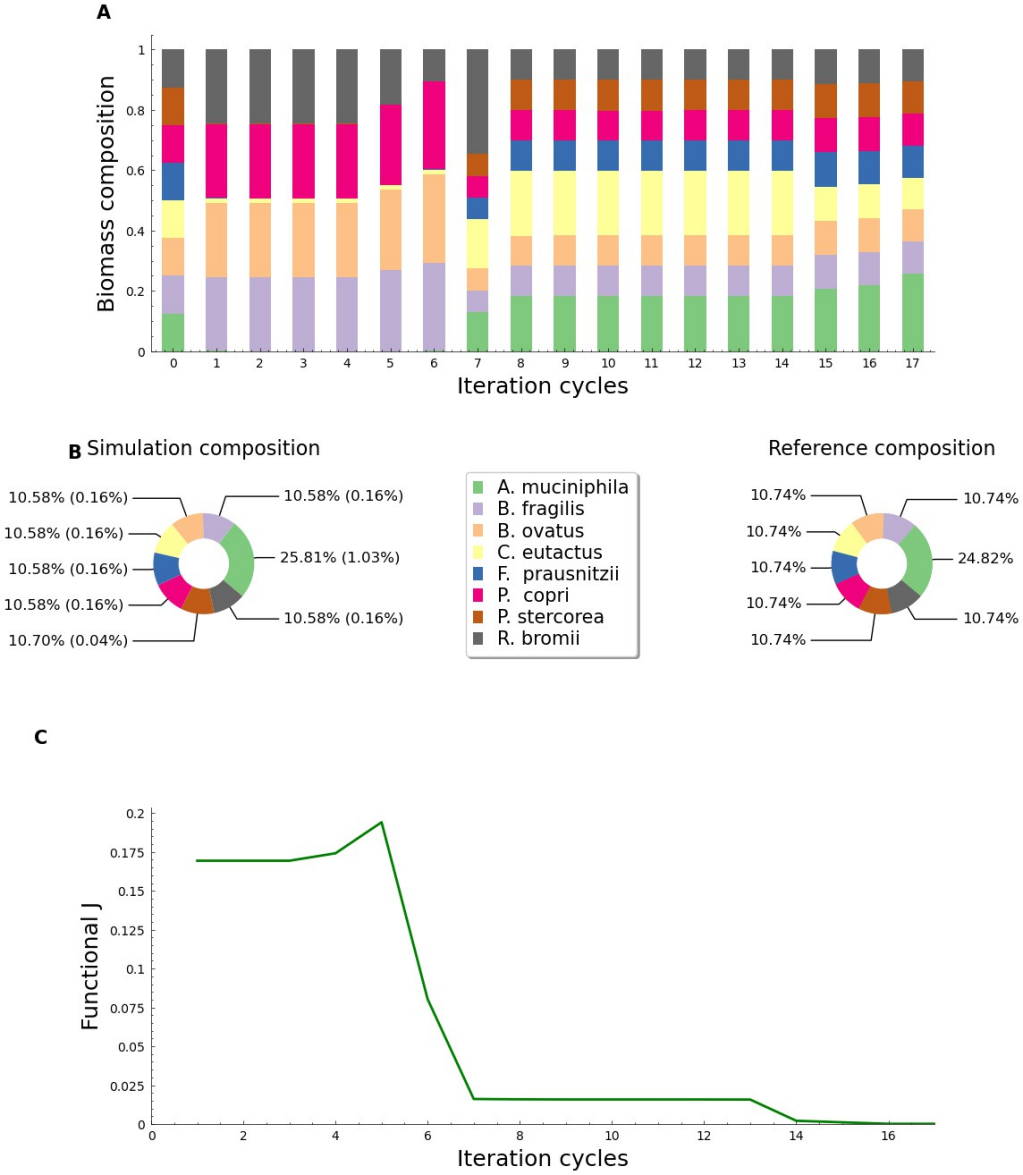
Evaluation of the optimization algorithm using mock microbiome reference values. A: Evolution of fraction community distribution concerning the cycles of adaptation enforced by the gradient descendant algorithm using a value of *α* = 0.125 and a mock microbiome composition; B: Comparison of pie charts for the mock reference biomass percentage distributions (left) and the obtained with the modeling strategy based on the gradient descent algorithm (right). C: Performance functional (*J*) evolution for the mock microbiome simulation.

Initially, we established initial reference values for a representative sample of an eight-member microbiome community with an initial arbitrary distribution where all reference values were the same for all species. We also selected the nutrition parameters inside the model script. After one hundred iterations, we obtained the reference values used as a reference for the optimization algorithm (Section 2.3). As shown in Fig 2A, the convergence of the community distribution towards the reference values was achieved after seventeen cycles of iteration. The method’s effectiveness, as indicated by the weighted error (w.e.), was found to be 0.2% for most species, except *A. muciniphila*, which had a w.e value five times higher (Fig 2B). Additionally, the average error percentage ($\bar{w.e}$) for all species was 0.25% with a standard deviation (SD) value of 1.015. The numerical simulation was concluded by reaching the early stop criterion value of J ≤ 0.001 (Fig 2C). The function exhibits a monotonically decreasing behavior at cycles 5–7, which is consistent with the change in distribution shown in Fig 2A at the same cycle stage.

### 3.2 Performance of the modeling scheme when using complex microbial distributions

**Fig 3:**
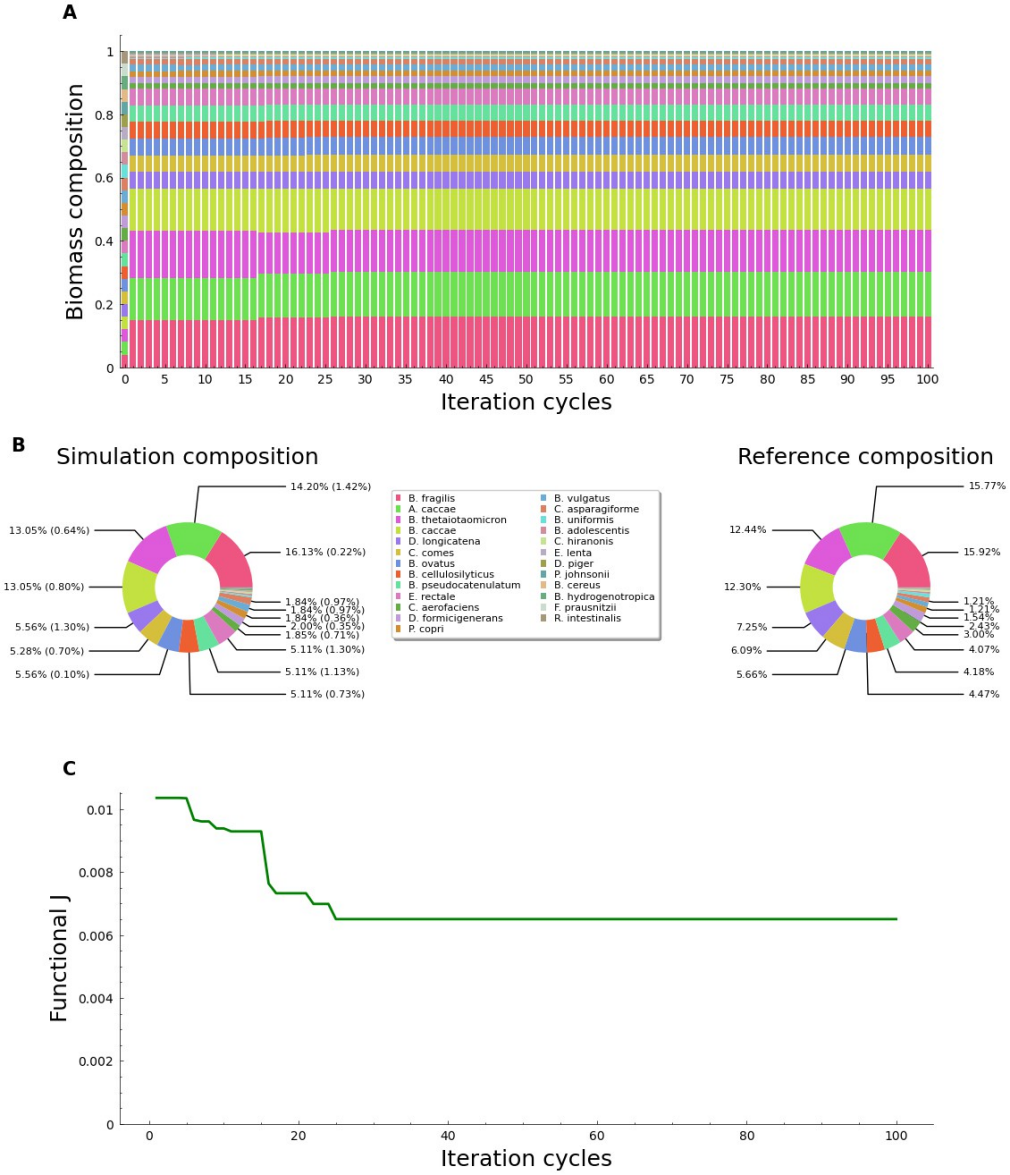
Evaluation of the optimization algorithm using *in vitro* microbiome reference values. A: Evolution of biomass fraction distribution during the cycles of adaptation enforced by the gradient descent algorithm using a value of *α* = 0.25 and *in vitro* abundance data obtained from [21]; B: Comparison of pie charts for the *in vitro* reference biomass percentage distributions (left) and the obtained with the modeling strategy based on the gradient descent algorithm (right); C: Performance functional (*J*) evolution for *in vitro* microbiome simulation.

For this section, we tested the nested algorithm’s ability to replicate *in vitro* and clinical bacterial distributions (Section 2.3). The results for the former show that after eighteen cycles of adaptation using the optimization algorithm, the distribution converges towards the *in vitro* experimental distribution values (Fig 3A). The final distribution was estimated with a $\bar{w.e}$ of 0.53% and an SD of 0.46 with a maximum specific w.e of 1.41% corresponding to *A. caccae* (Fig 3B). The performance functional (J) remained constant at a value of 0.032 after 25 cycles (Fig 3C), which meant that the early stop criterion was not fulfilled and indicating that the optimization algorithm reached a local minimum. Nevertheless, after the manual stop of the algorithm, the reported values were sufficiently low to consider the simulation adequate.

**Fig 4:**
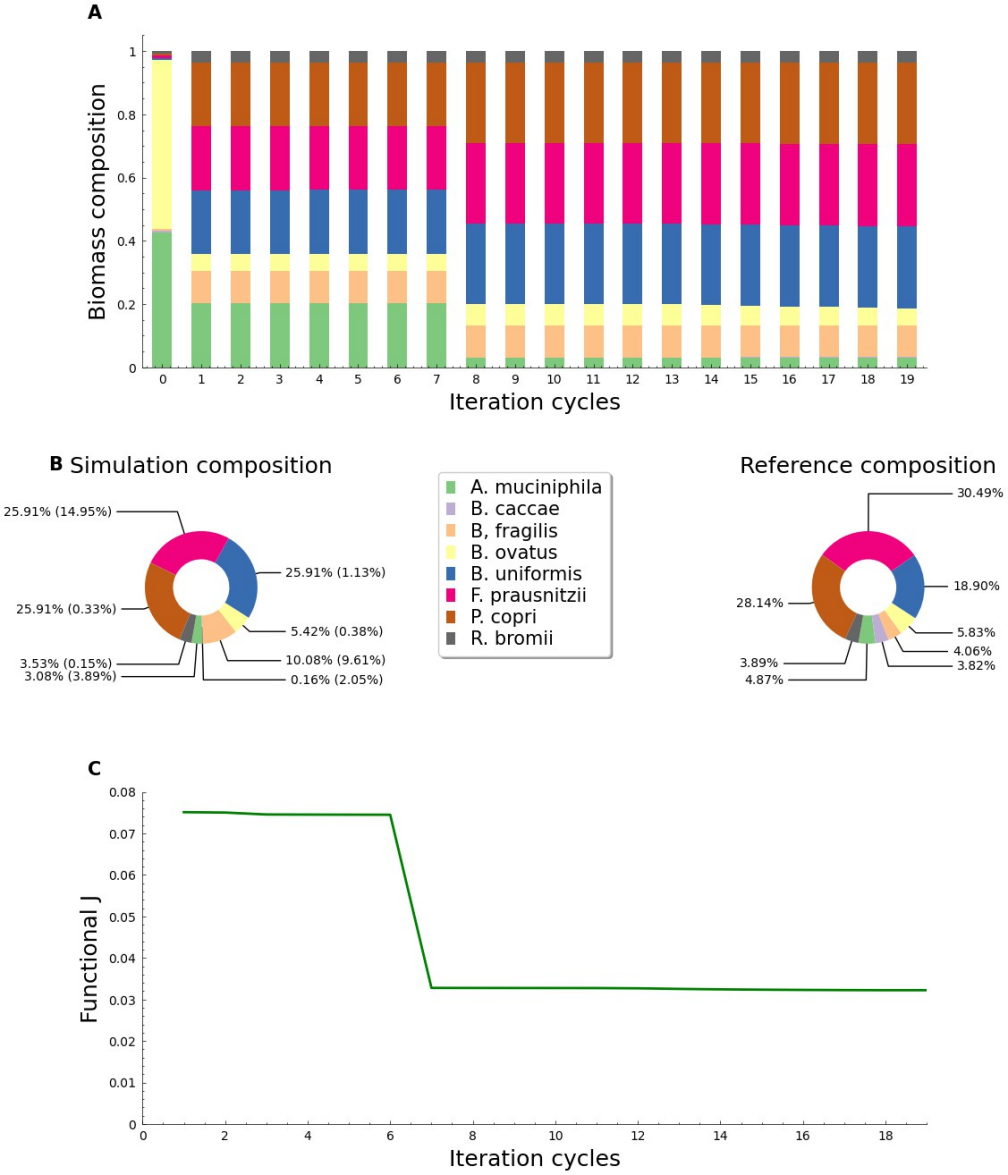
Evaluation of the optimization algorithm using clinical microbiome reference values. A: Evolution of biomass fraction distribution for the cycles of adaptation enforced by the gradient descendant algorithm using a value of *α* = 0.125 and a microbiome composition from Mexican patients published by [20]; B: Comparison of pie charts for the clinical reference biomass percentage distributions (left) and the obtained with the modeling strategy based on the gradient descent algorithm (right); C: Performance functional (*J*) evolution using clinical data from healthy Mexican patients for the simulation.

In the case of the clinical data, exploratory modeling experiments demonstrated that *A. muciniphila* and *B. caccae* did not change their biomass concentration in the dynamic simulation process. This could indicate that these strains do not interact with other community members in the specified conditions. Hence, the initial biomass concentrations for these strains were fixed to the values obtained during the exploratory experiment. The evolution of the microbial distribution after this change was done is shown in Fig 4A. After 15 recursive steps of the optimization algorithm, the biomass fractions in the microbial consortium were estimated with with a maximum specific error (w.e) of 14.95% (Fig 4B). The $\bar{w.e}$ for the simulation amounted to 4.0% and a standard deviation of 5.4. Similarly to the previous test, the variation of J after 15 steps is almost zero, confirming the convergence of the functional to a local minimum (Fig 4C). When comparing the results to the obtained in the *in vitro* scenario, there seems to be an increase in the error percentage. This could be attributed to the complexity when trying to interpret clinical data compared to a designed community with well-defined and validated data. *In vivo* external factors that influence microbiome composition are numerous and challenging to quantify [3]. Thus, the proposed method does not involve parameter estimation as part of the simulation results. Model contextualization is crucial for obtaining parameters that closely match those observed in natural and *in vitro* settings. However, using identical kinetic parameters values across all compounds and species means that the results are not intended for biological interpretation and should be interpreted as generic, as specified by [14].

### 3.3 Metabolite composition estimation for the three data conditions

**Fig 5:**
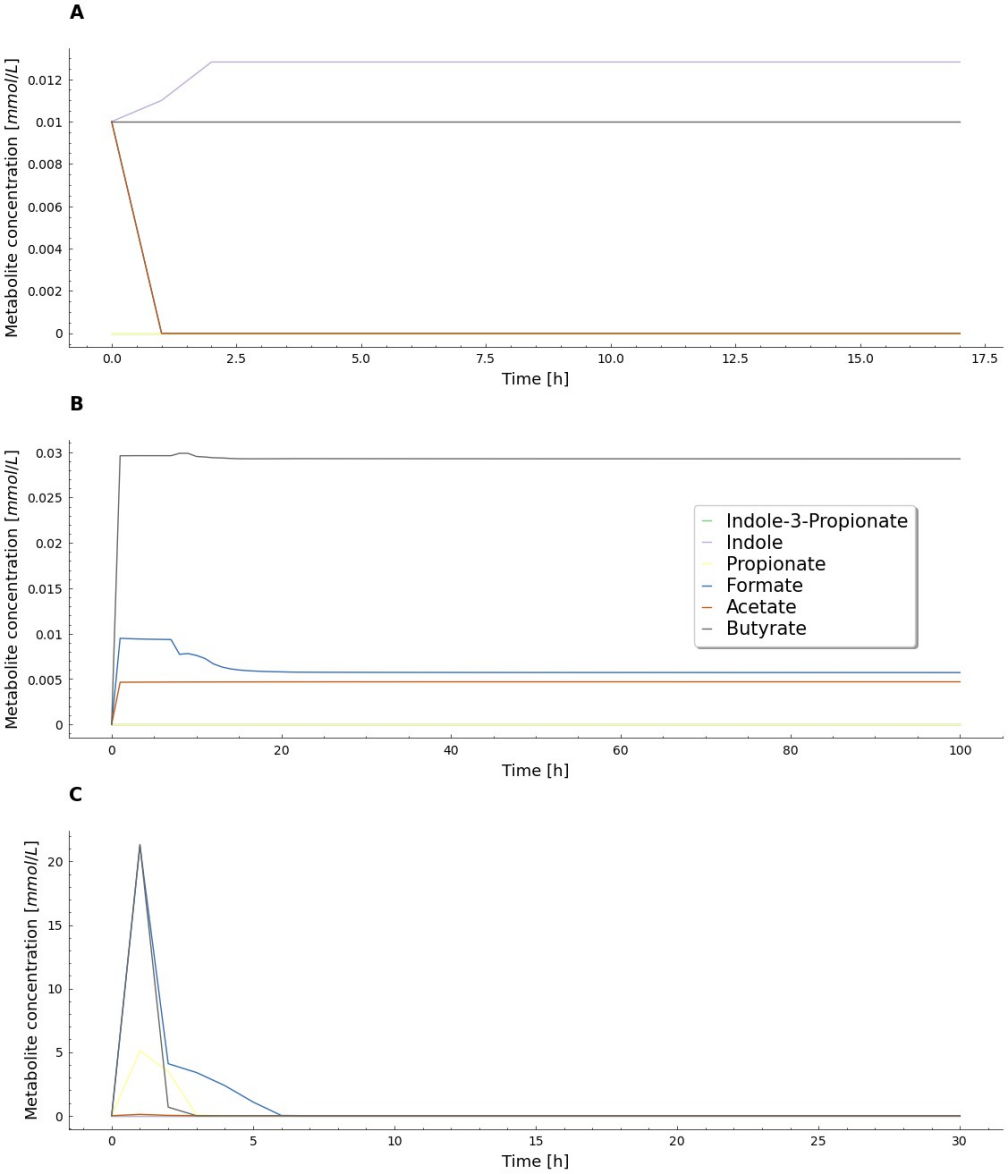
Temporal evolution of SCFA produced along the last iteration of the gradient descendant algorithm for each case. Mock microbiome (A), *in vitro* microbiome (B) and clinical microbiome (C) community distributions.

The metabolic reconstructions used for the modeling scheme enabled a temporal analysis of the dynamics of secondary metabolites that varied when the gradient descent strategy was iterated. At the end of the optimization process for bacterial distribution in all datasets, the evolution of selected metabolites was observed (Fig 5). The presented results provide a condensed representation of the benefits of employing GEM methodologies to study changes in metabolite profiles in microbial communities.

Analyzing the mock community results, the rapid formation and consumption of metabolites, as shown in Fig 5, can be explained as follows. The compounds in the nutritional space were initializez in the simulations with a concentration of 0.01 mM for each compound. The selected *K*_*m*_ value (0.01 mM) represents a high affinity of the microorganisms for these compounds, resulting in rapid consumption of substrates. SCFA-producing bacteria such as *B. fragilis* and *B. ovatus* were included in the simulation, and their abundance percentage in the mock community (Fig 2A) may explain the presence of indole in the metabolite pool, as these bacteria produce indole due to the presence of the enzyme tryptophanase in their genome [25].

Additionally, other SCFAs such as acetate, butyrate, and formate are produced by bacterial species such as *A. muciniphila*, *F. prausnitzii*, *R. Bromii* and others [26, 27] which correlates with the compound concentrations observed in the *in vitro* results Fig 5B). The clinical case exhibits the formation of butyrate, formate, and propionate, but their levels were quickly depleted. The production of these short-chain fatty acids (SCFAs) in the community simulation were expected as a result of the known production by *F. prausnitzii* and *A. muciniphila* [28–32]. Recent studies have demonstrated that bacterial species can engage in cross-feeding of SCFAs, particularlly in environments with limited or no carbohydrate supply [33]. This cross-feeding phenomenon could account for the observed consumption of these metabolites in Fig 5C and 5C. For the rest of the metabolites, no significant changes were observed in all simulated cases.

## 4 Discussion

Microbiome compositions have been linked to affecting host health and disease [35]. The complexity of these microbial communities and the multiple factors influencing species distribution pose the main challenge when trying to understand their effects in the host ecosystem [6]. Currently, *in silico* ecological models typically describe interactions as a function of population abundances but provide no information regarding the metabolic status of species. The main contribution of the presented work is the implementation of gradient descent as a way to modify kinetic parameters in a dFBA simulation to replicate the bacterial relative abundance composition from different sources. μbialSim was selected as a numerical simulator for the features mentioned in Section 2.1. However, the simulation employs the Augmented Euler method to solve ordinary differential equations, which is particularly useful in situations with high numbers of consuming species or high compound affinities. This can result in smaller time steps to maintain accuracy, making the simulation more computationally demanding and, in some cases, infeasible to solve. Alternative dFBA simulators, such as DFABlab [35, 36], COMETS [15], or SurfinFBA [37], could have been applied using the proposed methodology. While these alternatives offer new features, they might require modifications as some operate using a different programming language.

The modeling strategy considered in this study allowed us to: (1) include individual dynamic relationships and metabolite interchange between microbial species within the system’s framework; (2) reproduce *in vitro* and clinical microbiome compositions in terms of their relative abundance effectively; (3) observe key metabolite evolution inside the nutritional space of the system. For instance, in our studies, the community compositions were replicated with a maximum weighted average error of 4% in clinical data. This certainty value comes from implementing the hierarchical gradient descent algorithm, and the value was in an acceptable range for error. Moreover, the numerical simulator implemented in this strategy allowed us to observe changes in the concentration of selected metabolites, which are important for inter-species and host interactions. While this depends directly on the chosen numerical simulator, the fact that we can observe these changes implies a complete integration of the optimization algorithm and the toolboxes. However, the results found in this work should be verified through experimental investigations.

The bacterial metabolic reconstructions can be further contextualized to better fit the studied system. A mechanistic understanding of microbiome communities is limited to the information available about the system [6]. This limitation also applies to the dynamic community simulator [14], which requires kinetic parameters that are difficult to estimate experimentally. The dynamic parameter estimation approach employed in this study consistently reproduces the reference abundance distribution in all the cases. This demonstrates the robustness and accuracy of the strategy. However, it is important to note that these results should only be considered biologically meaningful after conducting controlled experiments to verify the findings. In our specific case, the datasets used in the paper draft were previously published by [20, 21]. Both of the cited sources fail to provide specific information on dynamic parameters related to the bacterial strains under investigation. However, it is important to acknowledge that neither study primarily addresses the evaluation of bacterial growth. However, it is important to note that bacterial growth is successfully simulated in our study. By employing the optimization algorithm to adjust parameter values, such as Vmax, the community is able to attain reference abundance compositions. Thus, leading to a considerable understanding of the general bacterial interactions taking place to reach that abundance composition. However, the outlined procedure in this study remains applicable if additional experimental data becomes available. Current studies address these issues by using non-structured modeling techniques (e.g., gLV), which simplify the data needed for these to work [38–41].

The presented approach overcomes some limitations regarding the need for a significant amount of data to observe microbial behavior and interaction. The improvement lies in the combination of the mathematical model from the μbialSim numerical simulator and the modeling strategy based on the gradient descent algorithm, which is known for ts ability to work with relatively small amounts of data, as confirmed by our results. Lower error percentages are attributed to taking only the fractional composition of the distribution as a measure of error between the simulation and the reference values. This implies that the applicability of our strategy will improve as more kinetic experimental data becomes available.

Dynamic modeling strategies employ different numerical methods to infer the kinetic parameters for the model [42–44]. The traditional least means square regression algorithm, commonly used for its simplicity, infers kinetic parameters after multiple cycles have passed, which limits its applicability when using such models for decision-making [45]. The gradient descent algorithm used in this work is an optimization algorithm that works in line with the simulation, modifying the parameters on each cycle. Some studies employ machine learning algorithms to predict community structures for various diseases using metagenomic data [46]. However, this leads to a more reductionist understanding of the underlying metabolic interactions in the system. In addition to the above facts, most of those studies require many samples to predict diseases depending on microbial compositions correctly.

The current work paves the road for a new modeling approach that complements GEM modeling strategies’ current state-of-the-art. We expect this modeling strategy for microbiome communities to be useful in facilitating the interpretation of metagenomic sequencing data in the context of metabolic interactions, mainly to study systems for which samples are hard to acquire. Moreover, *in silico* determination of the change of metabolites within the microbiome environment may help us to understand better the relation with the host and the alteration of the immune system, which is highly influenced by microbiome changes [5, 47–52]. For example, we managed to evidence this phenomenon by observing the evolution of the metabolic production and consumption in the simulations, therefore, confirming the presence of microbial interactions. The metabolic profile is therefore directly related to the bacterial composition, as our results showed. However, these results still require experimental validation [53, 54].

In conclusion, the combination of genome-scale metabolic modeling and the gradient descent optimization algorithm to infer bacterial kinetic and metabolic behavior provides promising opportunities for studying the gut microbiome and its interaction with the host without the need for extensive data. However, the accuracy of the simulation results is contingent upon the comprehensiveness of the model system description. Our tool is publicly available and expected to undergo further continuous development. The strategy is scalable and applicable to different GEM-based technologies. Potentially, it is feasible that the modeling technique can be scalable to different applications, such as the evaluation of external components to the nutritional space (e.g. the effects of pharmaceuticals) in the microbial dynamics, especially when *in vitro* experiments with complex microbiome compositions become challenging in laboratory settings.

## Supporting information

S1 Table Chemically defined medium “DMR38”, conversion to mmol. (XLSX)

S1 Dataset Last iteration trajectory files for each abundance case. (ZIP)
